# Systematic review, meta-analysis of cusp-overlap vs. three-cusp coplanar approaches in self-expandable transcatheter aortic valve replacement

**DOI:** 10.3389/fcvm.2026.1870774

**Published:** 2026-07-02

**Authors:** Mohammad Ghannam, Mustafa Abomohsen, Iyad Y. Idries, Fayez Shamoon, Rahul Vasudev, Khaled Moghib, Muhammad Ghallab, Fawzi Zghyer, Alena V. González, Abdullah Ahmad, Habib A. Habib

**Affiliations:** 1 Brookdale University Hospital and Medical Center, Brooklyn, NY, USA; 2 St. Joseph’s University Medical Center, Paterson, NJ, United States; 3 Faculty of Medicine Kasralainy Cairo University, Cairo, Egypt; 4 Department of Cardiovascular Medicine, Creighton University, Omaha, NE, United States; 5 Heart, Vascular and Thoracic Institute, Cleveland Clinic Foundation, Cleveland, OH, United States; 6 Department of Cardiology, Heart Failure Division, University of Alabama at Birmingham, Birmingham, AL, United States

**Keywords:** cerebrovascular accident, conduction disturbances, cusp-overlap, mortality, permanent pacemaker implantation, transcatheter aortic valve replacement

## Abstract

**Background:**

The cusp-overlap technique (COT) is an alternative fluoroscopic approach to the standard three-cusp technique (ST) in self-expandable transcatheter aortic valve replacement (TAVR). It may improve implantation depth and reduce conduction disturbances, but its overall clinical benefit remains unclear.

**Objectives:**

To compare the safety and efficacy of COT vs. ST in terms of mortality, conduction disturbances, permanent pacemaker implantation (PPI), and cerebrovascular accidents (CVA).

**Methods:**

This systematic review and meta-analysis followed PRISMA guidelines. PubMed, Cochrane, Scopus, Embase, and Web of Science were searched up to October 2025. Studies comparing COT and ST were included. Data extraction and risk-of-bias assessment were performed independently. Meta-analysis was conducted using R with fixed- and random-effects models. Outcomes included mortality (by follow-up), conduction disturbances, PPI, and CVA. The protocol was registered in PROSPERO (CRD420251156402).

**Results:**

Eighteen studies were analyzed, with patient numbers varying by outcome (mortality: 5,984 patients; conduction disturbances: 7,200 patients; PPI: 5,712 patients; CVA: 4,066 patients). COT significantly reduced mortality compared with ST (OR: 0.60, 95% CI: 0.40–0.88; *p* = 0.010; *I*^2^ = 0%), with the strongest effect observed at 30 days. Conduction disturbances were also lower with COT (OR: 0.62, 95% CI: 0.48–0.82; *p* = 0.0006; *I*^2^ = 77.7%), driven by reductions in both complete atrioventricular block and left bundle branch block. The risk of PPI was markedly reduced with COT (OR: 0.50, 95% CI: 0.39–0.63; *p* < 0.0001; *I*^2^ = 23.0%). In contrast, there was no significant difference in CVA between groups (random-effects OR: 1.05, 95% CI: 0.73–1.51; *p* = 0.78; *I*^2^ = 0%).

**Conclusion:**

COT in self-expandable TAVR is associated with lower rates of conduction disturbances and PPI, and reduced short-term mortality, without increasing cerebrovascular risk. While these findings are consistent and clinically plausible, the overall certainty of evidence is limited. COT appears to be a promising procedural refinement, but further randomized studies are needed to confirm long-term benefits.

**Systematic Review Registration:**

identifier CRD420251156402.

## 1 Introduction

Severe aortic stenosis represents one of the most prevalent and life-threatening valvular heart diseases in the aging population, with a rapidly increasing global burden driven by demographic shifts toward older age groups (1–3). Over the past decade, transcatheter aortic valve replacement (TAVR) has transformed the management paradigm for patients across the entire surgical-risk spectrum, evolving from a therapy reserved for inoperable individuals to a first-line treatment option in many contemporary clinical scenarios (4, 5). Despite remarkable advances in device design and procedural refinement, conduction disturbances remain among the most frequent and clinically consequential complications after TAVR, particularly with self-expandable valve systems (6, 7). New-onset left bundle branch block, complete atrioventricular block, and the subsequent need for permanent pacemaker implantation (PPI) are consistently associated with prolonged hospitalization, impaired ventricular function, and adverse long-term outcomes, underscoring the persistent unmet need for procedural strategies that can mitigate injury to the atrioventricular conduction system (4, 5, 8).

**Figure 1:**
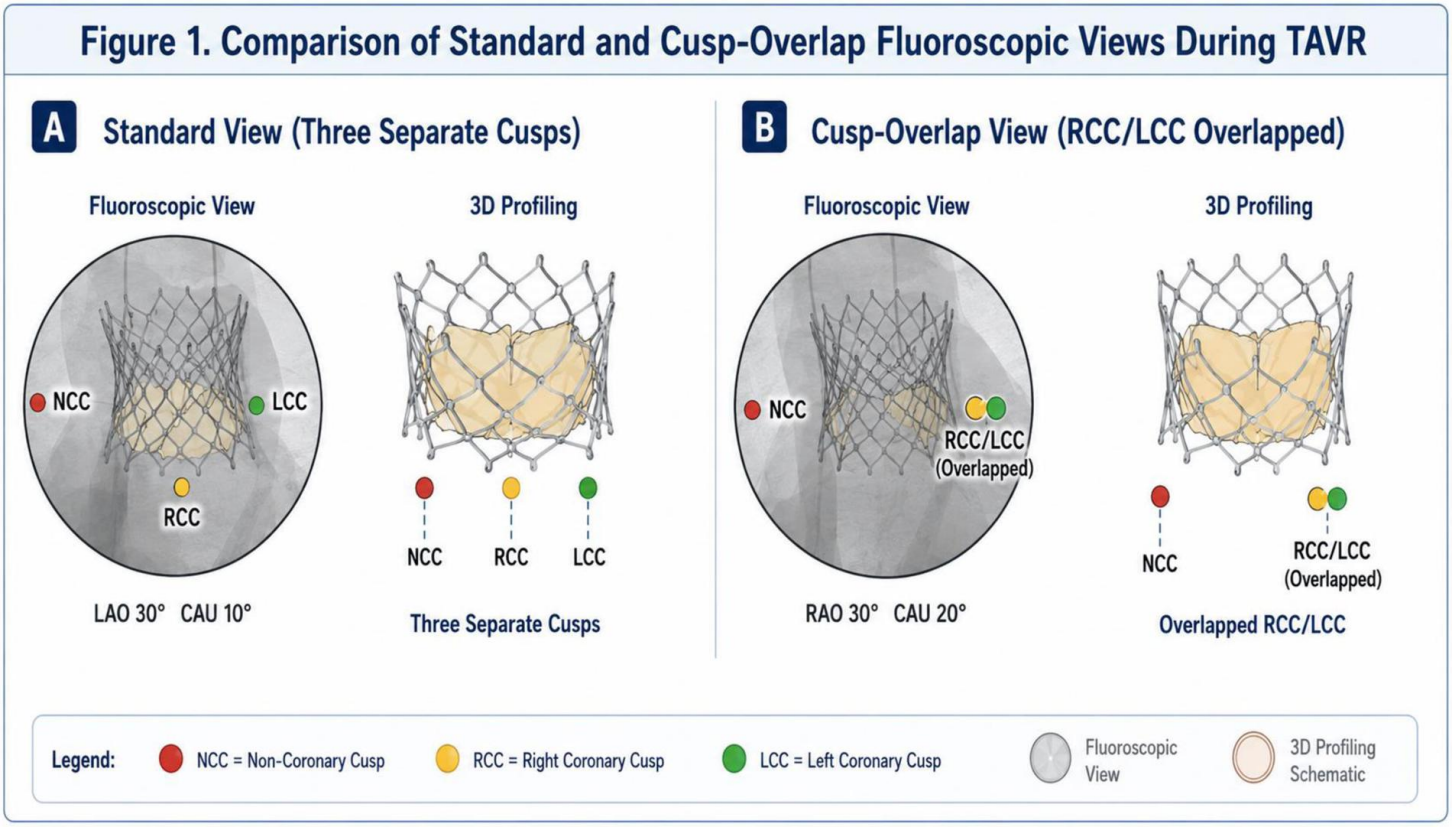
Comparison of standard and cusp-overlap fluoroscopic views during TAVR. **(A)** Standard View: Fluoroscopic projection demonstrating the conventional three-cusp orientation, with the NCC, RCC, and LCC visualized separately. Corresponding 3D profiling shows distinct cusp alignment. **(B)** Cusp-Overlap View: Fluoroscopic projection obtained using a cusp-overlap orientation, in which the RCC and LCC are aligned and overlapped, leaving the NCC isolated. Corresponding 3D profiling demonstrates the overlapped RCC/LCC configuration, facilitating optimized valve deployment assessment. Created in BioRender. Moghib (2026) https://BioRender.com/y2rz1t0, licensed under Academic License.

The depth and orientation of valve implantation have emerged as critical, modifiable determinants of post-procedural conduction disturbances in self-expandable TAVR. Traditionally, implantation is guided using the standard three-cusp coplanar fluoroscopic projection, which aligns the right, left, and non-coronary cusps in a single plane (9, 10). Although widely adopted, this projection may lead to systematic underestimation of true implantation depth relative to the non-coronary cusp, potentially increasing mechanical interaction between the prosthesis frame and the muscular septum (11, 12). In recent years, an alternative fluoroscopic strategy, the cusp-overlap technique, has been proposed to improve visualization of the left ventricular outflow tract and enhance control of deployment depth by overlapping the right and left coronary cusps while isolating the non-coronary cusp. Mechanistically, this approach is intended to facilitate higher and more precise valve positioning, thereby reducing compression of the conduction tissue and subsequent electrical complications (9, 10, 13) (Figure 1).

Several observational studies and registries have suggested that the cusp-overlap technique may be associated with lower rates of complete atrioventricular block and permanent pacemaker implantation when compared with the standard three-cusp approach in self-expandable TAVR. However, the available evidence remains observational and heterogeneous, with substantial variation in study size, patient populations, valve platforms, operator experience, follow-up duration, and outcome definitions (14–16). As a result, individual studies may be underpowered to detect clinically important differences in less frequent outcomes such as mortality and cerebrovascular events. Although pooling observational evidence cannot eliminate the inherent limitations of non-randomized research, systematic review and meta-analysis can improve the precision of effect estimates, provide a more comprehensive assessment of consistency across studies, and identify important sources of between-study heterogeneity. Consequently, uncertainty remains regarding the overall safety and effectiveness of cusp-overlap-guided implantation and the extent to which its reported benefits translate into meaningful clinical outcomes.

As the cusp-overlap technique is increasingly adopted in routine practice and incorporated into training programs, a comprehensive synthesis of the rapidly expanding literature is critically needed. To date, no comprehensive evidence synthesis has systematically integrated contemporary comparative data evaluating the cusp-overlap technique in self-expandable TAVR. Although several observational studies have reported favorable procedural and clinical outcomes, the consistency and magnitude of these effects across different patient populations and clinical settings remain uncertain. Therefore, a rigorous systematic review and meta-analysis is needed to provide a more precise estimate of the safety and effectiveness of the cusp-overlap technique and to inform contemporary clinical practice (17–19).

Therefore, we conducted a systematic review and meta-analysis to comprehensively compare the cusp-overlap technique with the standard three-cusp coplanar approach in patients undergoing self-expandable TAVR. The primary objectives were to evaluate differences in all-cause mortality, conduction disturbances, including complete atrioventricular block and left bundle branch block**,** permanent pacemaker implantation, and cerebrovascular accidents. By integrating all available comparative evidence and formally investigating sources of between-study variability, this review aims to provide a robust and clinically meaningful assessment of the true impact of cusp-overlap–guided implantation and to clarify its role as a procedural strategy for improving outcomes in contemporary self-expandable TAVR practice.

## 2 Methods

This study was conducted following the Cochrane Handbook for Systematic Reviews of Interventions (20). The results were reported as specified by the Preferred Reporting Items for Systematic Reviews and Meta-Analyses (PRISMA) statement (21). Additionally, this study has been registered with PROSPERO under the identification number (CRD420251156402).

### 2.1 Information sources and search strategy

A comprehensive literature search was conducted across five electronic databases, namely Cochrane Library, PubMed, Embase, Web of Science, and Scopus, from inception to October 30, 2025. The search strategy combined controlled vocabulary and free-text terms related to transcatheter aortic valve replacement and imaging techniques, including [“Transcatheter Aortic Valve Replacement” (Mesh) OR TAVR OR TAVI OR transcatheter aortic valve implantation] AND [“Aortic Valve Stenosis” (Mesh) OR aortic stenosis OR aortic valve disease] AND (cusp overlap OR cusp-overlap view OR overlap view OR three-cusp OR three-cusp view OR coplanar view) AND (self-expandable OR self-expanding OR SE valve OR CoreValve OR Evolut) for more details (see Supplementary Appendix Table 1), together with relevant synonyms. Two reviewers independently and concurrently performed the database searches. In addition to electronic database searches, the reference lists of all eligible studies and relevant review articles were manually screened to identify potentially relevant studies that may not have been captured by the predefined search strategy. No additional eligible studies were identified through this process.

### 2.2 Eligibility criteria

#### 2.2.1 Inclusion criteria

Observational studies enrolling patients with aortic stenosis who underwent transcatheter aortic valve replacement using self-expanding valves and directly comparing the cusp-overlap view technique with the conventional three-cusp view technique were eligible for inclusion. Studies were required to report at least one outcome related to procedural performance, effectiveness, or safety. Eligible outcomes included procedure time, technical and procedural success according to VARC-3 definitions, need for permanent pacemaker implantation, requirement for a second valve implantation, and adverse events, including new-onset left bundle branch block, moderate or severe aortic regurgitation, valve embolization, cerebrovascular accidents, complete atrioventricular block, aortic annular rupture, paravalvular leak, major bleeding, major vascular complications, and all-cause mortality.

#### 2.2.2 Exclusion criteria

Studies were excluded if they were not published in English; were review articles, meta-analyses, theses, case reports, or case series; were animal or experimental studies; were technical reports or conference abstracts without available full texts; or evaluated valve types other than self-expanding devices, including balloon-expandable or mechanically expandable valves.

#### 2.2.3 Screening and data management

The screening process was carried out in two distinct phases. The first phase involved screening titles and abstracts to identify potentially relevant studies. In the second phase, full-text articles of selected studies were thoroughly reviewed to confirm eligibility. Rayyan software was used to facilitate the screening process. Two independent reviewers conducted the eligibility assessment, and any disagreements were resolved by consulting a third reviewer (22). Duplicate records identified during database searches were removed using Rayyan software and verified manually. During full-text review, studies were also assessed for potential overlap in patient populations by comparing participating centers, recruitment periods, registry sources, and study characteristics. When overlapping cohorts were suspected, the study with the most comprehensive dataset, largest sample size, or longest follow-up duration was retained, while duplicate or partially overlapping reports were excluded to avoid double-counting of participants.

### 2.3 Data extraction

Four reviewers independently extracted data from all eligible studies for the cusp-overlap and conventional three-cusp groups using standardized online extraction sheets, and any discrepancies were resolved by consensus through group discussion. The collected data included study-level characteristics (study design, country, population, intervention and comparator, follow-up duration, eligibility criteria, and authors' conclusions), baseline participant characteristics (mean age, proportion of males, smoking status, and the prevalence of dyslipidemia, diabetes mellitus, hypertension, peripheral artery disease, transient ischemic attack, and prior myocardial infarction), procedural characteristics (access approach, use of pre- and post-dilation, valve pop-out, valve type, and valve size), primary outcomes (mortality, post-procedural conduction disturbances, permanent pacemaker implantation, bleeding, and cerebrovascular events), and secondary outcomes (vascular complications, moderate or severe aortic regurgitation, valve embolization, annular rupture, and paravalvular leak).

### 2.4 Risk of bias assessment

Two independent reviewers conducted a risk of bias assessment for the studies included in the analysis. For cohort and non-randomized studies of interventions, the Risk Of Bias In Non-randomized Studies, of Interventions (ROBINS-I) assessment tool was applied (23, 24). This tool assesses seven domains: bias due to confounding, bias in selection of participants, bias in classification of interventions, bias due to deviations from intended interventions, bias due to missing data, bias in measurement of outcomes, and bias in selection of the reported result. Based on this comprehensive assessment, each study was rated as having low, moderate, serious, or critical risk of bias, and an overall risk of bias judgment was assigned accordingly.

### 2.5 Publication bias

To assess publication bias, several statistical methods were employed. The Fail-Safe N test was performed using the Rosenthal approach to determine the number of additional null studies required to render the overall meta-analysis results nonsignificant. Begg and Mazumdar's rank correlation test was applied to evaluate the relationship between effect sizes and their standard errors, detecting potential asymmetry in the funnel plot. Egger's regression test was utilized to assess the linear relationship between effect size and precision, providing further insight into funnel plot asymmetry. These methods collectively provided a comprehensive evaluation of potential publication bias in the analysis (25–27).

### 2.6 Choice of the meta-analysis model

The pooled effect sizes across all outcomes were calculated using the Mantel–Haenszel method to estimate odds ratios within a random-effects framework, with between-study variance (*τ*^2^) estimated using the restricted maximum-likelihood (REML) approach. This model accounts for potential heterogeneity among studies by incorporating between-study variability into the pooled estimate, resulting in wider and more conservative confidence intervals. Therefore, the results should be interpreted with consideration of possible residual inconsistency across the included studies (28).

### 2.7 Calculation of missing data

In instances where data were reported as the median and interquartile ranges (IQR), conversions were performed to obtain mean (M) and standard deviation (SD) utilizing the equations established by Wan et al. (29). Furthermore, when the standard deviation was not supplied, it was derived from the standard error using the formula for a single sample: SD = SE*√*n*, where (*n*) denotes the sample size (30). If the mean change (MC) between baseline and endpoint data was unavailable, it was calculated as MC = Mpost-treatment − Mpre-treatment. Additionally, in cases where the standard deviation of the mean change was not provided, it was computed from the standard deviations of the pre-treatment and post-treatment samples using the formula SD = √(SD^2^pre-treatment + SD^2^post-treatment) (31). Effect sizes (*d*) and effect size correlations (*r*) that were missing were calculated through the following formulas: *d* = (Mtreatment − Mcontrol)/SDpooled, with SDpooled calculated as √[(SD^2^treatment + SD^2^control)/2] (22), and *r* = *d*/√(*d*^2^ + 4) (32).

### 2.8 Statistical analysis

Heterogeneity across studies was assessed through visual inspection of forest plots and by calculating the *I*^2^ statistic and Cochran's *Q* test. Significant heterogeneity was defined as a Chi-Square *p* < 0.1 or *I*^2^ > 50%. In instances of significant heterogeneity, sensitivity analyses were performed by sequentially excluding individual studies to identify potential sources of inconsistency. All statistical analyses were conducted using R programming version 4.4.1 for Windows (33).

## 3 Results

### 3.1 Search and screening

**Figure 2:**
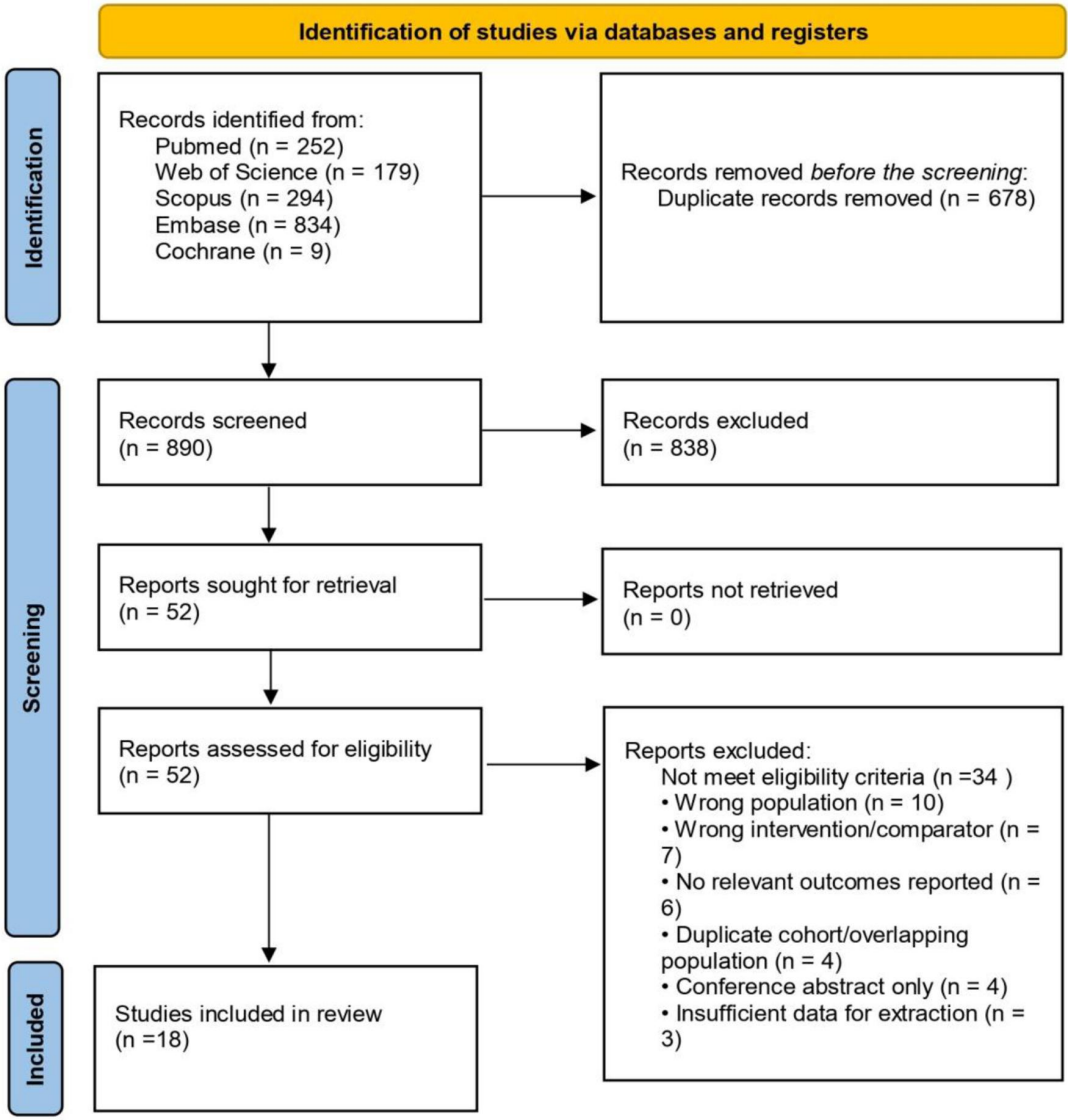
PRISMA flow diagram.

We identified 1,568 records through an initial search of the electronic databases (nine in Cochrane Library, 834 in Embase, 252 in PubMed, 294 in Scopus, and 179 in Web of Science), which were reduced to 890 articles after removing duplicates. Fifty- two articles satisfied the inclusion criteria during the title and abstract screening, and finally, after full-text screening, eighteen studies (34–51) were identified for inclusion in this systematic review and meta-analysis (Figure 2).

### 3.2 Studies’ characteristics

The pooled evidence comprised 18 primary reports from North America, Europe, Asia, and South America, the majority of which were retrospective cohort studies, with a minority of prospective series [e.g., (37, 38, 42)]. Study populations were patients undergoing transfemoral transcatheter aortic valve replacement (TAVR) with contemporary self-expanding devices (predominantly the Medtronic Evolut R/PRO family; several studies also included Portico and other SE valves). Inclusion criteria were broadly similar and generally defined symptomatic severe native aortic stenosis treated via transfemoral access; common exclusion criteria included prior permanent pacemaker implantation, valve-in-valve procedures, bicuspid aortic valves, and non-transfemoral approaches. Follow-up intervals varied by study and outcome: most studies reported periprocedural or 30-day endpoints (commonly used across 30-day analyses), several used in-hospital endpoints, one study reported outcomes at one week (51), and a minority reported longer (one-year) outcomes. Definitions of outcomes and conduction endpoints were not uniform, with studies using VARC-2 or VARC-3 criteria where reported, while some studies did not specify formal VARC definitions (noted as “NA”), contributing to methodological heterogeneity. Procedural characteristics were consistent in showing near-universal transfemoral access (>90% in most cohorts) and predominance of self-expanding valve sizes in the 26–29 mm range, although device selection and size distribution varied between centres. Use of pre- and post-dilatation differed across series (pre-dilation rates ranged widely, and post-dilation was variably reported), and valve pop-out events were rare. Across the included studies, most authors reported that the cusp-overlap technique (COT) facilitated higher implantation depth and an optimized valve position and was associated in many cohorts with lower rates of new conduction disturbances and reduced need for permanent pacemaker implantation; however, several reports [e.g., (40, 41)] found no statistically significant reduction in PPI. Notably, Lee et al. (51) demonstrated significantly lower rates of both new-onset left bundle branch block (27.7% vs. 46.0%, *p* = 0.006) and PPI (4.3% vs. 18.0%, *p* = 0.033) with COT, further supporting the technique's benefit, though the study was limited by its small sample size (*n* = 97) and 1-week follow-up duration. Taken together, these characteristics highlight consistent real-world application of COT with self-expanding valves but also important between-study differences in device mix, endpoint definitions, and follow-up duration that should be considered when interpreting pooled estimates (Tables 1, 2).

**Table 1:** Studies, characteristics.

Study ID	Study design	Country	Inclusion criteria	Exclusion criteria	Follow-up duration cusp overlap technique	Follow-up duration conventional technique	Definitions	Study conclusion
Aljabbary et al. (47)	Retrospective cohort	Canada	Patients who underwent a TAVR procedure with SE Evolut R/Pro valves using the cusp-overlap technique from July 2018 to September 2020	Patients who died during TAVR procedure	30 days	30 days	NA	The COT approach offers several potential technical advantages compared to standard three-cusp view, and may result in lower PPI rates in TAVR with SE Evolut valve.
Asmarats et al. (46)	Retrospective Cohort	Spain	Patients who underwent TAVR using standard three-cusp technique from April 2016 to March 2017	Patients who had a PPI before the TAVR procedure	30 days	30 days	VARC-3 (46)	Combination of cusp-overlap view and ICE achieved a reliably higher valve position with the substantial reduction in the rates of PPI and conduction disturbances without compromising procedural safety and valve hemodynamics.
Datta et al. (45)	Retrospective cohort	India	Patients undergoing transfemoral TAVR with the self-expanding Portico FlexNav system	Patients with previous PPI	In hospital	In hospital	VARC-3 (45)	COT technique during Portico FlexNav implantation is feasible and facilitates a higher valve implant, which in turn may help to reduce rates of new-onset conduction disturbances.
Doldi et al. (44)	Retrospective cohort	Germany	Patients with symptomatic severe aortic stenosis who underwent TAVR with a SE Core Valve Evolut R between January 2017 and September 2022 and completed follow-up data at 90 days.	Patients who underwent TAVR with a balloon-expandable valve. Patients who had a PPI prior to TAVR. Patients with bicuspid aortic valves.	90 days	90 days	VARC-3 (45)	The study concluded that the COT is associated with a lower incidence of PPI and new-onset LBBB compared to the conventional coplanar technique in patients undergoing TAVR with a SE Core Valve Evolut R.
Güzel et al. (43)	Retrospective cohort	Turkey	patients with severe symptomatic aortic stenosis Patients at high risk for surgical aortic valve replacement, defined as an STS score ≥ 4 or a logistic EuroSCORE II ≥ 10% Patients who were able to provide informed consent	Patients with prior surgical bioprosthetic aortic valve replacement or prior TAVR Patients with prior PPI Patients who were not able to provide informed consent	1 year	1 year	VARC-3 (46)	The study concluded that the use of the COT for TAVR with the SE CoreValve Evolut prosthesis may be associated with a lower risk of PPI compared to the traditional three-cusp view technique. The COP technique may help to reduce the conduction disturbances, PPI requirement and complication rates that may develop following TAVR. In addition, it is an interesting result that it reduces mortality and MACCE rates in long- term follow-ups.
Ishizu et al. (42)	Prospective cohort	Japan	patients who underwent elective or emergency transfemoral TAVR with a self-expandable CoreValve Evolut valve between January 2015 and March 2022 patients who were eligible candidates for TAVR	Patients who had PPI or had a history of surgical aortic valve replacement, TAVR, balloon-expandable TAVR, valve-in-valve procedure, or bicuspid aortic valves TAVR with a balloon-expandable valve Previous cardiac electronic device	30 days	30 days	VARC-3 (45)	The study concluded that the use of the COT for TAVR with the SE CoreValve Evolut prosthesis may be associated with a lower risk of PPI compared to the traditional three-cusp view technique.
Lee et al. (51)	Retrospective cohort	Taiwan	AS patients who underwent TAVR with SEV (Medtronic CoreValve Evolut R or Evolut PRO) from Jan 2019 to Jan 2022	Pre-existing pacemaker; valve-in-valve procedures	1 week	1 week	VARC-2	The COL technique significantly reduced newly developed LBBB and PPI after SEV TAVR. Keeping SEV implantation depth within 1 mm of the membranous septum length and maintaining <6 mm depth below the LCC before final release further minimizes PPI risk.
Maier et al. (50)	Retrospective cohort	Germany	patients who underwent transfemoral TAVR with newer-generation self-expandable CoreValve Evolut system	Most of the patients excluded from the final analysis due to missing data have lack of documentation in procedural characteristics (pre- and post- dilatation, resheathing, valve dislocation) or post-procedural evaluation of valve function by missing documentation of echocardiography	30 days	30 days	VARC-2 (46)	Transcatheter aortic valve replacement using the COT is associated with an optimized implantation depth, leading to fewer permanent conduction disturbances.
Medranda et al. (40)	Retrospective cohort	USA	patients with symptomatic severe tricuspid AS who underwent native aortic valve TAVR using the third-generation self-expanding CoreValve Evolut PRO/PRO+	previous PPI or ICD	In hospital	In hospital	VARC-3 (46)	The COT may not yield a reduction in PPI rates when compared with the coplanar technique.
Mendiz et al. (39)	Retrospective cohort	Argentina	Patients underwent elective or emergent TAVR with COT with selfexpandable valves	Patients with bicuspid aortic valve, having previous PPI, receiving balloon-expandable valve implantation or other self-expandable valves (Acurate Neo and Portico) and those who underwent a valve-invalve procedure	30 days	30 days	VARC-2 (45)	The COT decreases the 30-day LBBB and PPI rate.
Mendiz et al. (49)	Retrospective cohort	Argentina	patients receiving TAVR with selfexpandable valves from August 2019 to May 2021	Patients with surgical bioprosthesis, prior PPI, bicuspid aortic valve or TAVR with balloon-expandable valve	30 days	30 days	VARC-2 (45)	High implantation of the percutaneous aortic valve using the COT was feasible and safe, with lower need for PPI at 30 days and a lower incidence of new-onset LBBB.
Moura et al. (41)	Retrospective cohort	Portugal	patients underwent TAVR in the context of severe native aortic valve disease with self-expanding new generation CoreValve Evolut R or Evolut PRO between April 18, 2019 and November 22, 2021	Patients with a previously implanted PPI, cardiac resynchronization therapy device or ICD, bicuspid aortic valve, or who underwent valve-in-valve procedures	30 days	30 days	VARC-2 (45)	This study shows that the COT, although safe and not associated with increased complexity, did not significantly reduce the 30-day PPI rate compared to the traditional view.
Pascual et al. (38)	prospective study	Spain	patients with severe symptomatic AS who underwent TAVR from 28 March 2017 to 12 November 2020	Patients who underwent TAVR with other valve type, valve in valve procedure, PPI, different femoral access	30 days	30 days	VARC-2 (45)	The COT provides a higher implantation of self-expanding THV with lower rates of conduction disturbances and PPI
Pascual et al. (37)	Prospective study	Spain, Canada, Mexico	Patients who underwent transcatheter aortic valve replacement (TAVR) using the Medtronic Evolut self-expanding (SE) valve.	Patients who were unable to provide informed consent, underwent nontransfemoral approach or had previous PPI, age < 18 years, valve-in-valve procedures.	30 days	30 days	VARC-2 (45)	The COT significantly reduced the rate of PPI compared to the classical implantation technique. COT optimized the implantation depth (ID), leading to better outcomes
Persia-Paulino et al. (36)	Retrospective cohort	Spain	Patients undergoing TAVI with self-expandable valves selected by a multidisciplinary heart team	Patients with PPI, bicuspid aortic valve disease, unreadable electrocardiograms (ECG) prior to TAVR, atrial fibrillation, and previous valve-in-valve TAVI	1 year	1 year	NA	At 1 year of follow-up, COT reduced the incidence of new onset left bundle branch block. COT was also associated with a statistically significant reduction in the occurrence of the combined primary cardiovascular endpoint.
Sztejfman et al. (35)	Retrospective cohort	Argentina	All TAVR patients	patients with previous PPI	30 days	30 days	VARC-2 (45)	TAVR with the COT was associated with a null pacemaker requirement at 30 days, suggesting a potential benefit in avoiding pacemaker implantation post-TAVR
Takaseya et al. (34)	Retrospective cohort	Japan	Patients with native severe aortic stenosis who underwent TAVR with a self-expandable TAVR via the transfemoral approach	Patients with previous cardiac electronic devices, bicuspid aortic valves, or severe complications such as left ventricular perforation, valve migration requiring repeat valve implantation, or severe low cardiac output syndrome requiring mechanical assistance after TAVR	30 days	30 days	NA	TAVR using the COT is associated with optimized implantation depth, leading to fewer conduction disturbances and reduced paravalvular leakage (PVL)
Wienemann et al. (48)	Retrospective cohort	Germany	Patients who underwent TAVR with the Evolut R, PRO, or PRO + between February 2016 and April 2022.	Patients under 18 years of age, those with prior surgical or transcatheter aortic valve replacement, and prior PPI	In hospital	In hospital	NA	The introduction of the COT was associated with a significant reduction in rates of PPI and paravalvular regurgitation without an increase in complication rates

**Table 2:** Procedural characteristics.

Study ID	Technique	Femoral access	Subclavian access	Pre-dilation	Post-dilation	Valve pop-out	Valve type	Valve size (23 mm)	Valve size (26 mm)	Valve size (29 mm)	Valve size (34 mm)
Aljabbary et al. (47)	Cusp Overlap	515 (99.2)	3 (0.6)	270 (52)	99 (19)	2 (0.4)	Evolut R/PRO	31 (6)	188 (36)	235 (45)	65 (12.5)
	Conventional	126 (98.4)	2 (1.6)	26 (20)	28 (22)	3 (2.3)		10 (8)	43 (33.6)	58 (45)	2 (1.6)
Asmarats et al. (46)	Cusp Overlap	NA	NA	32 (76.2)	13 (31.0)	NA	Portico	10 (23.8)	NA	5 (11.9)	NA
	Conventional	NA	NA	37 (86.1)	20 (46.5)	NA		14 (32.6)	NA	4 (9.3)	NA
Datta et al. (45)	Cusp Overlap	55 (98)	1 (1.8)	19 (33.9)	18 (32.14)	2 (3.6)	Evolut R or PRO	NA	NA	NA	NA
	Conventional	53 (96.4)	2 (3.6)	21 (38.8)	15 (27.3)	2 (3.6)		NA	NA	NA	NA
Doldi et al. (44)	Cusp Overlap	61 (100)	0 (0)	NA	NA	NA	Evolut R	5 (8.2)	32 (52.5)	22 (36.1)	2 (3.3)
	Conventional	61 (100)	0 (0)	NA	NA	NA		8 (13.3)	33 (55.0)	19 (31.7)	0 (0.0)
Güzel et al. (43)	Cusp Overlap	105 (100)	0 (0)	38 (36.2)	30 (28.6)	NA	Evolut	NA	NA	NA	NA
	Conventional	176 (100)	0 (0)	42 (23.9)	40 (22.7)	NA		NA	NA	NA	NA
Ishizu et al. (42)	Cusp Overlap	223 (94.9)	NA	233 (99.1)	51 (21.7)	NA	Evolut R/PRO/PRO+	40 (17)	143 (60.9)	46 (19.6)	6 (2.6)
	Conventional	188 (89.1)	NA	206 (97.6)	41 (19.4)	NA		35 (16.6)	124 (58.8)	52 (24.6)	0 (0)
Lee et al. (51)	Cusp Overlap	42 (89.4)	5 (10.6)	9 (19.1)	22 (46.8)	NA	Evolut PRO (91.5%), Evolut R (8.5%)	NA	NA	NA	2 (4.3)
	Conventional	48 (96.0)	2 (4.0)	11 (22.0)	23 (46.0)	NA	Evolut R (92%), Evolut PRO (8%)	NA	NA	NA	2 (4.0)
Maier et al. (50)	Cusp Overlap	150 (100)	0 (0)	71 (47.3)	23 (15.3)	2 (0.1)	Evolut	2 (1.3)	36 (24.0)	73 (48.7)	38 (25.3)
	Conventional	150 (100)	0 (0)	65 (43.3)	19 (12.7)	2 (0.1)		2 (1.3)	37 (24.7)	64 (42.7)	47 (31.3)
Medranda et al. (40)	Cusp Overlap	258 (100)	0 (0)	NA	NA	NA	Evolut PRO/PRO+	NA	NA	NA	NA
	Conventional	270 (100)	0 (0)	NA	NA	NA		NA	NA	NA	NA
Mendiz et al. (39)	Cusp Overlap	156 (100)	0 (0)	86 (55.1)	34 (21.8)	1 (0.64)	Evolut R/PRO	7 (4.5)	39 (25)	75 (48.1)	35 (22.4)
	Conventional	101 (100)	0 (0)	58 (57.4)	25 (24.5)	0 (0)		3 (3)	18 (17.8)	57 (56.4)	22 (21.8)
Mendiz et al. (49)	Cusp Overlap	61 (95.2)	2 (3.17)	50 (79.3)	16 (25.3)	NA	Evolut R/PRO, Portico, Acurate neo	NA	7 (11.1)	26 (41.3)	18 (28.6)
	Conventional	101 (100)	NA	58 (57.4)	25 (24.5)	NA		3 (3)	18 (17.8)	58 (57.4)	22 (21.4)
Moura et al. (41)	Cusp Overlap	60 (100)	NA	43 (71.7)	24 (40.0)	NA	Evolut R or PRO	NA	NA	NA	NA
	Conventional	60 (96.8)	NA	22 (35.5)	31 (50.0)	NA		NA	NA	NA	NA
Pascual et al. (38)	Cusp Overlap	113 (100)	0 (0)	13 (11.50)	33 (29.20)	NA	Evolut	0 (0)	24 (21.24)	57 (50.44)	32 (28.32)
	Conventional	113 (100)	0 (0)	21 (18.58)	45 (39.82)	NA		3 (2.65)	30 (26.55)	49 (43.36)	31 (27.43)
Pascual et al. (37)	Cusp Overlap	161 (100)	0 (0)	24 (14.9)	43 (26.7)	NA	Evolut	1 (0.6)	45 (28.0)	78 (48.5)	37 (23)
	Conventional	161 (100)	0 (0)	31 (19.3)	53 (32.9)	NA		6 (3.7)	47 (29.2)	78 (48.4)	30 (18.6)
Persia-Paulino et al. (36)	Cusp Overlap	NA	NA	13 (14.1)	27 (29.3)	NA	NA	NA	NA	NA	NA
	Conventional	NA	NA	18 (19.6)	36 (39.1)	NA		NA	NA	NA	NA
Sztejfman et al. (35)	Cusp Overlap	15 (100)	0 (0)	NA	NA	NA	Evolut-R or PRO	NA	NA	NA	NA
	Conventional	50 (100)	0 (0)	NA	NA	NA		NA	NA	NA	NA
Takaseya et al. (34)	Cusp Overlap	48 (100)	0 (0)	41 (85)	7 (15)	NA	Evolut PRO/PRO+	7 (15)	26 (54)	13 (27)	2 (4)
	Conventional	49 (100)	0 (0)	46 (94)	9 (18)	NA		4 (8)	25 (51)	18 (37)	2 (4)
Wienemann et al. (48)	Cusp Overlap	953 (95.8)	NA	561 (56.4)	285 (28.6)	NA	Evolut-R/PRO/PRO+	35 (3.5)	313 (31.5)	476 (47.8)	171 (17.2)
	Conventional	959 (96.4)	NA	394 (39.6)	267 (26.8)	NA		33 (3.3)	297 (29.8)	479 (48.1)	186 (18.7)

### 3.3 Risk of bias assessment

**Figure 3:**
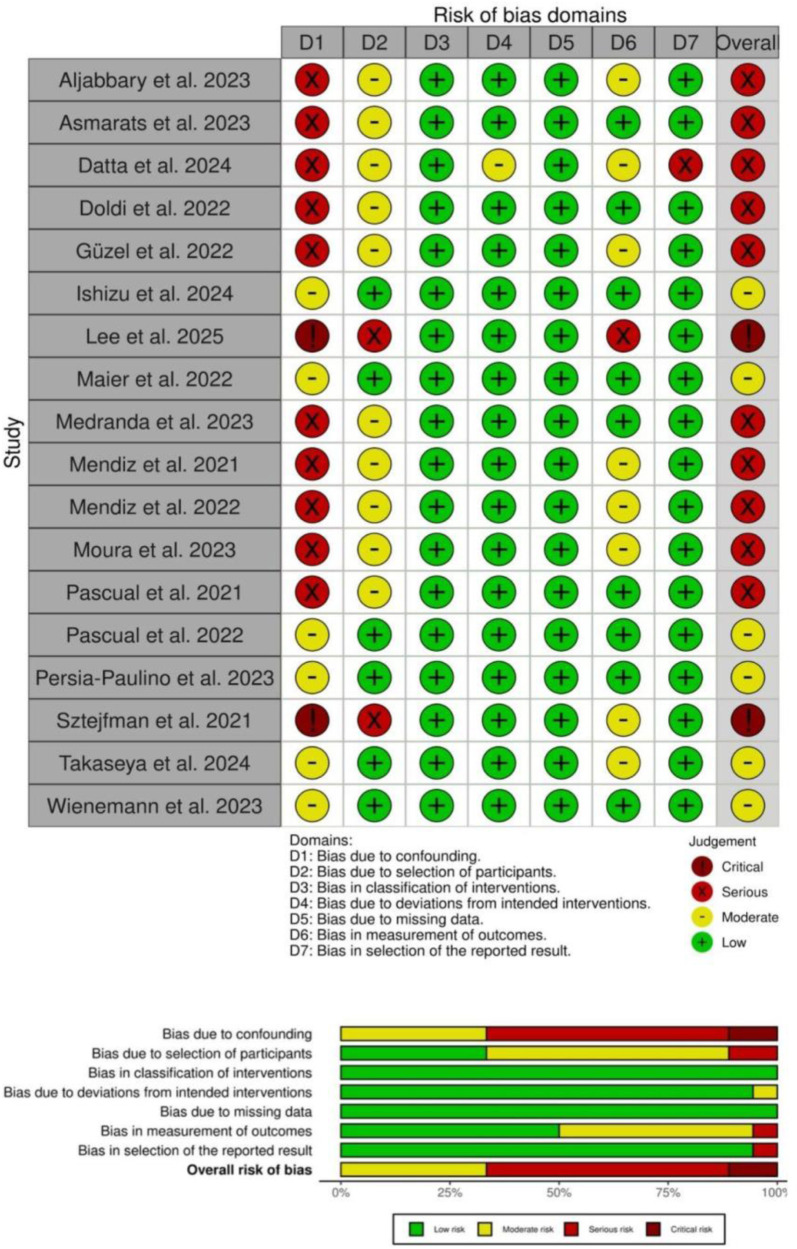
“Risk of bias graph”: review authors' judgments about each risk of bias item presented as percentages across all included studies, and “Risk of bias summary”: review authors’ judgments about each risk of bias item for each included study.

The risk of bias for the 18 included studies was assessed using the Risk Of Bias In Non-randomized Studies – of Interventions (ROBINS-I) tool. The overall risk of bias was rated as moderate in six studies, serious in ten studies, and critical in two studies (35, 51). No study achieved an overall low risk of bias. The most frequent limitation was bias due to confounding (D1), as all studies employed non-randomized, predominantly before-and-after designs, introducing temporal confounding from evolving operator experience, device iterations, and changes in PPI thresholds over time. Six studies mitigated this through propensity score matching or robust multivariable adjustment. Bias in measurement of outcomes (D6) varied depending on the use of standardized VARC criteria and protocol-driven PPI decisions. Bias in classification of interventions (D3), deviations from intended interventions (D4), and missing data (D5) were consistently low across studies. A detailed domain-by-domain assessment is provided in Figure 3.

### 3.4 Outcomes

#### 3.4.1 Mortality (in-hospital, 30-day, and one-year)

**Figure 4:**
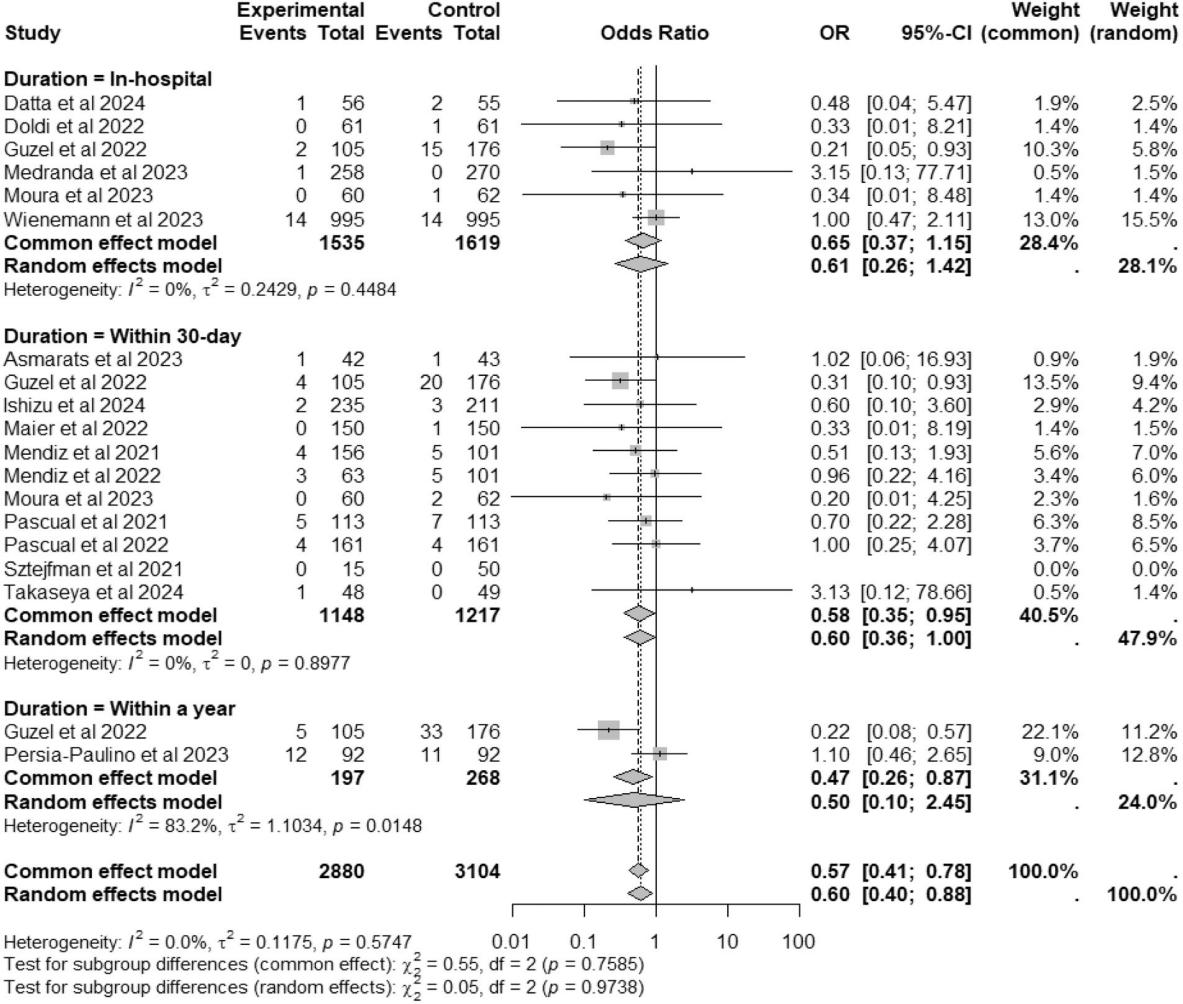
Forest plot showing in-hospital mortality, 30-day mortality, and one-year mortality when using COT vs. ST.

Eighteen studies comprising 5,984 patients (2,880 in the COT group and 3,104 in the ST group), with a total of 184 mortality events, were included in the pooled analysis. COT was associated with a statistically significant reduction in overall mortality compared with ST under both the common-effect model (OR: 0.57, 95% CI: 0.41–0.78; *p* = 0.0004) and the random-effects model (OR: 0.60, 95% CI: 0.40–0.88; *p* = 0.010). There was no evidence of statistical heterogeneity (*I*^2^ = 0%, *τ*^2^ = 0.1175; *p* = 0.57), indicating a high level of consistency across studies. Subgroup analysis based on follow-up duration showed that in-hospital mortality did not differ significantly between groups (random-effects OR: 0.61, 95% CI: 0.26–1.42), while 30-day mortality demonstrated a borderline reduction favoring COT (random-effects OR: 0.60, 95% CI: 0.36–1.00). One-year mortality showed a numerically lower risk with COT; however, this finding did not reach statistical significance (random-effects OR: 0.50, 95% CI: 0.10–2.45) and was accompanied by substantial heterogeneity (*I*^2^ = 83.2%). No statistically significant interaction was observed between follow-up durations (*p* for subgroup difference = 0.97), suggesting that the effect of COT on mortality was consistent over time (Figure 4).

#### 3.4.2 Conduction abnormalities (complete AV block and LBBB)

**Figure 5:**
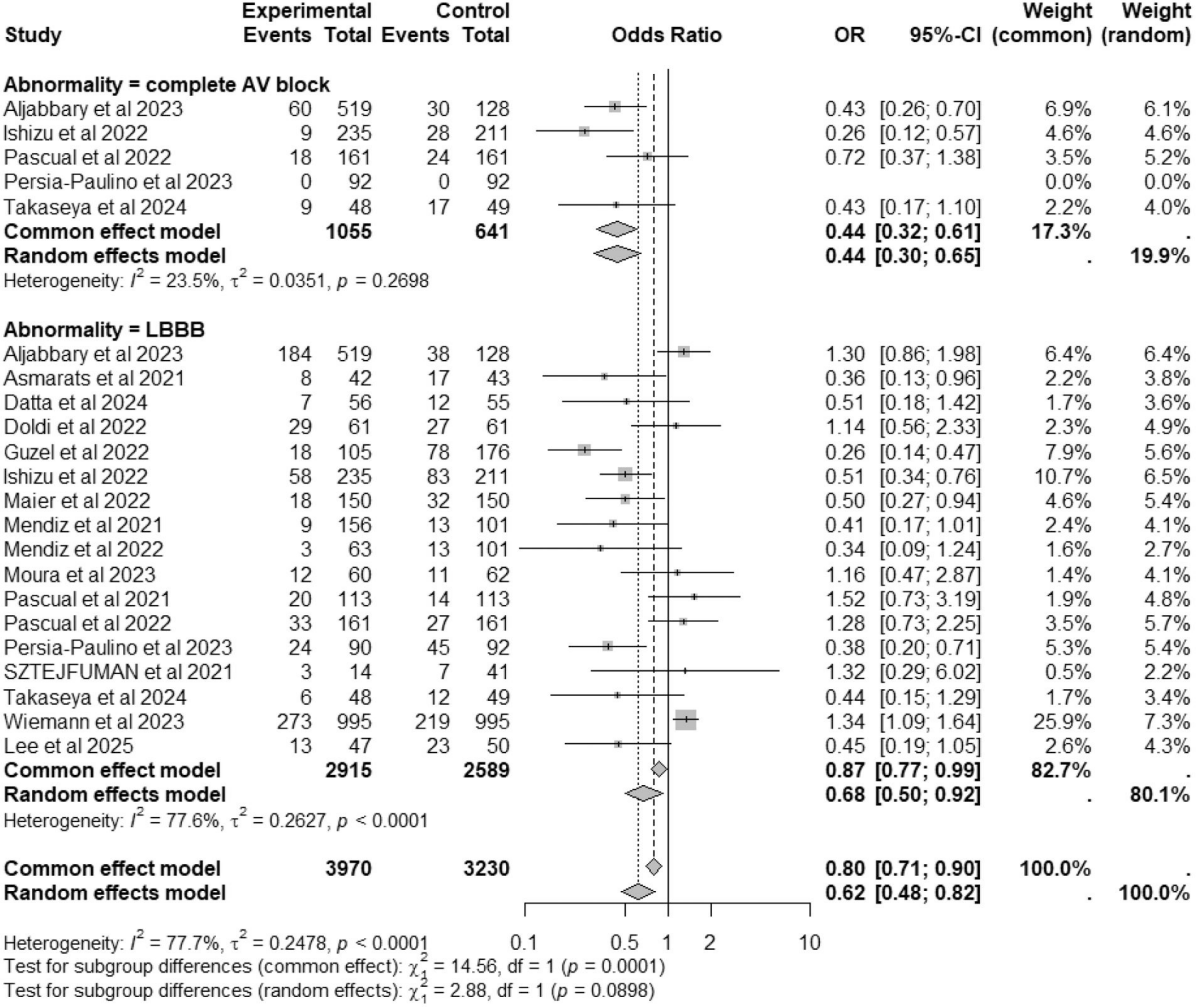
Forest plot showing complete AV block and LBBB when using COT vs. ST.

Sixteen studies including 7,200 patients, with 1,584 reported events, evaluated conduction abnormalities. Some studies contributed data to both the complete atrioventricular block and left bundle branch block subgroups, yielding 21 study-level comparisons. The pooled analysis demonstrated that COT was associated with a significant reduction in overall conduction disturbances compared with ST (random-effects OR: 0.62, 95% CI: 0.48–0.82; *p* = 0.0006). However, substantial heterogeneity was observed (*I*^2^ = 77.7%, *τ*^2^ = 0.2478; *p* < 0.0001), indicating variability in effect sizes across studies. Subgroup analysis revealed that COT significantly reduced the incidence of complete atrioventricular block (random-effects OR: 0.44, 95% CI: 0.30–0.65) with relatively low heterogeneity, while it also showed a modest but statistically significant reduction in left bundle branch block (random-effects OR: 0.68, 95% CI: 0.50–0.92), although heterogeneity remained high within this subgroup. The test for subgroup differences was not statistically significant under the random-effects model (*p* = 0.0898), suggesting that differences between specific types of conduction abnormalities should be interpreted with caution (Figure 5).

#### 3.4.3 Permanent pacemaker implantation (PPI)

**Figure 6:**
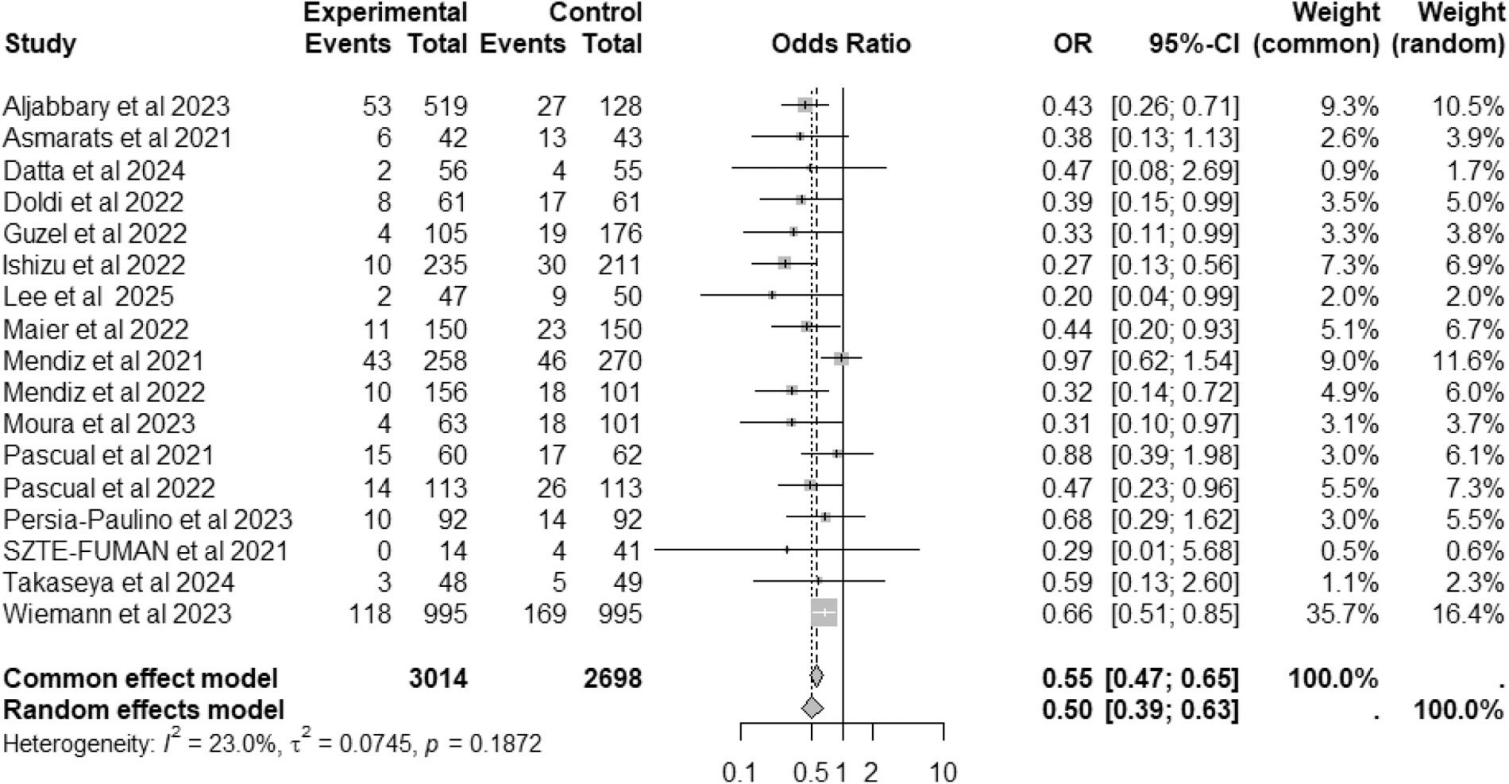
Forest plot showing PPI when using COT vs. ST.

Seventeen studies comprising 5,712 patients and 772 events reported outcomes related to permanent pacemaker implantation. The pooled analysis demonstrated that COT was associated with a significantly lower risk of PPI compared with ST under both the common-effect model (OR: 0.55, 95% CI: 0.47–0.65; *p* < 0.0001) and the random-effects model (OR: 0.50, 95% CI: 0.39–0.63; *p* < 0.0001). Heterogeneity was low (*I*^2^ = 23.0%, *τ*^2^ = 0.0745; *p* = 0.19), supporting the robustness and consistency of this finding across studies (Figure 6).

#### 3.4.4 Cerebrovascular accidents (stroke)

**Figure 7:**
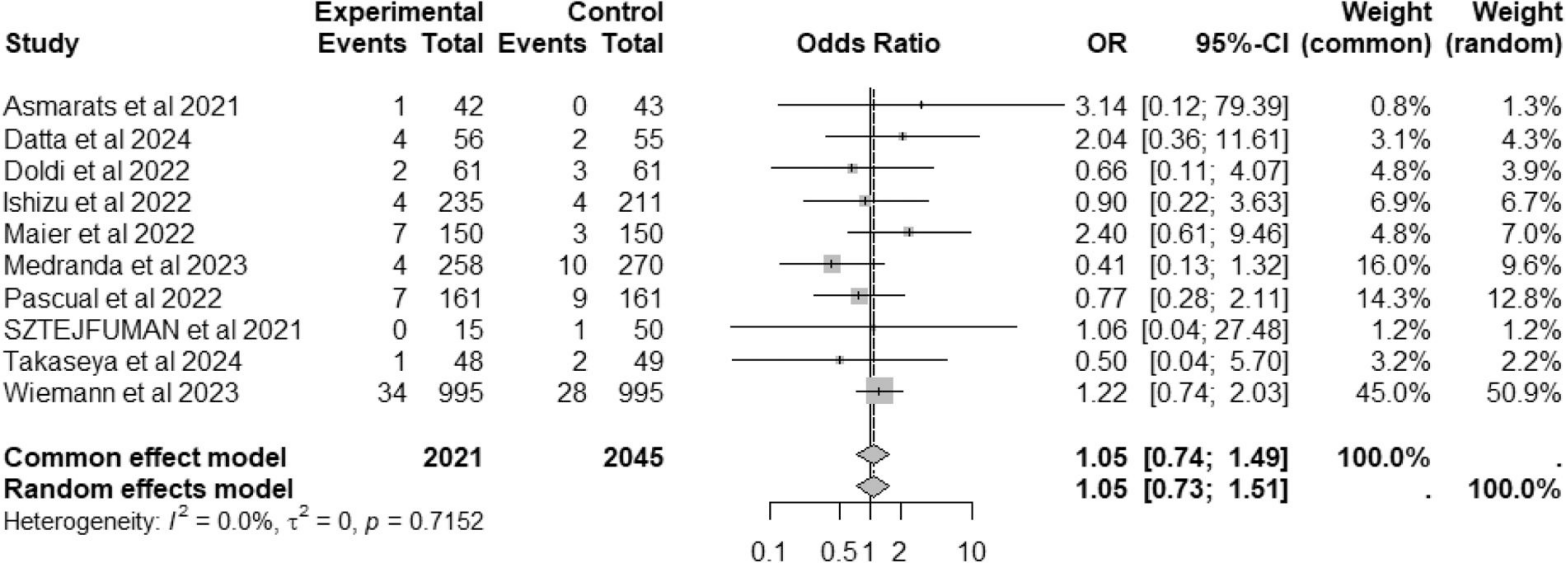
Forest plot showing cerebrovascular accidents when using COT vs. ST.

Ten studies, including 4,066 patients and 126 events, assessed the risk of cerebrovascular accidents. The pooled analysis showed no statistically significant difference between COT and ST (random-effects OR: 1.05, 95% CI: 0.73–1.51; *p* = 0.78). No heterogeneity was detected (*I*^2^ = 0%, *τ*^2^ = 0; *p* = 0.72), indicating highly consistent results across the included studies (Figure 7).

### 3.5 Publication bias

**Figure 8:**
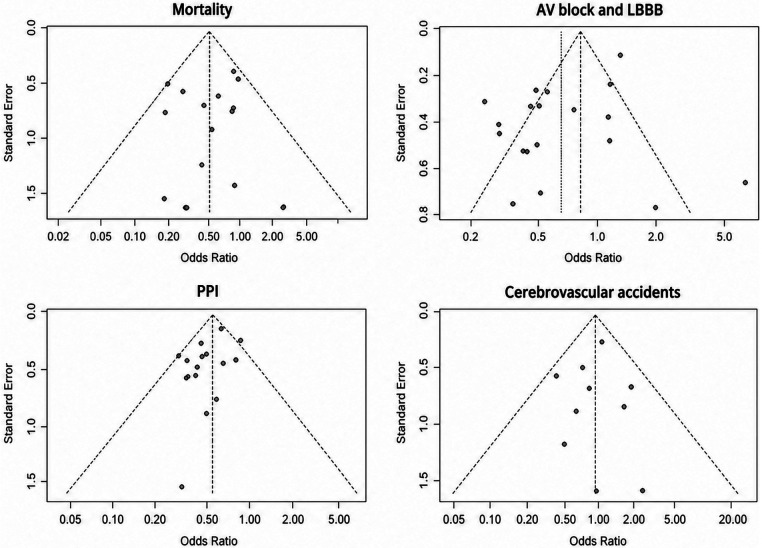
Funnel plots showing publication bias for 4 outcomes.

Publication bias was assessed using the trim-and-fill method. For the mortality outcome, two potentially missing studies were imputed, and the adjusted pooled estimate remained statistically significant (OR = 0.65, 95% CI: 0.44–0.96), indicating that the overall mortality benefit of COT over ST was robust to small-study effects. For conduction abnormalities, six studies were imputed; after adjustment, the effect estimate became non-significant (OR = 0.89, 95% CI: 0.60–1.31), suggesting the presence of potential publication bias and reduced certainty of the pooled effect. For permanent pacemaker implantation, six additional studies were imputed, and the protective effect of COT persisted (OR = 0.61, 95% CI: 0.48–0.77), indicating stability of the primary finding despite possible asymmetry. For cerebrovascular accidents, no studies were included, and the pooled estimate remained unchanged (OR = 1.05, 95% CI: 0.73–1.51), demonstrating no evidence of publication bias. Overall, trim-and-fill analyses suggested that the mortality and pacemaker outcomes were robust, whereas conduction abnormality results were more susceptible to small-study effects (Figure 8).

### 3.6 GRADE assessment

The certainty of evidence was evaluated using the Grading of Recommendations Assessment, Development and Evaluation (GRADE) framework. Because all included studies were observational and non-randomized, certainty ratings initially started at low certainty and were subsequently downgraded when concerns regarding risk of bias, inconsistency, imprecision, indirectness, or publication bias were identified.

For all-cause mortality, the certainty of evidence was rated as **low**. Although the pooled estimate demonstrated a significant reduction in mortality with the cusp-overlap technique, confidence was limited by the observational design of the included studies and the potential for residual confounding.

For conduction disturbances (complete atrioventricular block and left bundle branch block), the certainty of evidence was rated as **very low**. In addition to the inherent limitations of observational studies, substantial statistical heterogeneity was present (*I*^2^ = 77.7%), and trim-and-fill analyses suggested susceptibility to small-study effects and publication bias.

For permanent pacemaker implantation, the certainty of evidence was rated as **low**. The pooled effect was consistent across studies and heterogeneity was low; however, certainty remained limited by the observational study designs and residual confounding.

For cerebrovascular accidents, the certainty of evidence was rated as **low**. Although heterogeneity was negligible, the number of events was relatively small and the confidence interval included the possibility of both benefit and harm.

Overall, the certainty of evidence supporting cusp-overlap-guided implantation ranged from very low to low. Consequently, while the observed reductions in conduction disturbances and permanent pacemaker implantation appear clinically meaningful and biologically plausible, additional high-quality randomized controlled trials are required before definitive conclusions can be drawn. Although the magnitude and direction of effect were generally consistent across outcomes, the observational nature of the evidence base limits causal inference. Therefore, the pooled estimates should be interpreted as associations rather than definitive treatment effects (Table 3).

**Table 3:** GRADE evidence profile.

Outcome	No. of studies	Participants	Relative effect (OR, 95% CI)	Risk of bias	Inconsistency	Indirectness	Imprecision	Publication bias	Overall certainty
All-cause mortality	18	5,984	0.60 (0.40–0.88)	Serious	Not serious	Not serious	Not serious	Undetected	LOW ⊕⊕◯◯
Conduction disturbances (AV block/LBBB)	16	7,200	0.62 (0.48–0.82)	Serious	Serious (*I*^2^ = 77.7%)	Not serious	Not serious	Suspected	VERY LOW ⊕◯◯◯
Permanent pacemaker implantation	17	5,712	0.50 (0.39–0.63)	Serious	Not serious	Not serious	Not serious	Possible	LOW ⊕⊕◯◯
Cerebrovascular accident	10	4,066	1.05 (0.73–1.51)	Serious	Not serious	Not serious	Serious	Undetected	LOW ⊕⊕◯◯

## 4 Discussion

In this systematic review and meta-analysis, we synthesized contemporary comparative evidence evaluating the cusp-overlap technique (COT) vs. the conventional three-cusp coplanar technique (ST) in patients undergoing self-expandable transcatheter aortic valve replacement. Across 18 studies including over 7,200 patients, COT was associated with significant improvements in both electrical and clinical outcomes. Specifically, the technique was linked to lower overall mortality, fewer conduction disturbances, and a substantial reduction in permanent pacemaker implantation (PPI), while maintaining a neutral effect on cerebrovascular events. These findings support the concept that fluoroscopic implantation strategy represents a modifiable procedural determinant of outcomes in self-expanding TAVR, and that achieving higher implantation depth does not appear to compromise procedural safety.

The most consistent and clinically important finding was the nearly 50% reduction in PPI and the marked decrease in complete atrioventricular block with COT. This observation is biologically plausible. The cusp-overlap projection isolates the non-coronary cusp and improves visualization of the left ventricular outflow tract, enabling a more controlled and higher valve deployment. Because the atrioventricular conduction system courses within the membranous septum adjacent to the non-coronary cusp, deeper implantation increases radial compression and electrical injury. By facilitating shallower implantation, COT likely reduces mechanical trauma to the conduction tissue and thereby decreases high-grade conduction block. Notably, the inclusion of Lee et al. (51) in our updated analysis contributed to a statistically significant reduction in left bundle branch block (random-effects OR: 0.68, 95% CI: 0.50–0.92), suggesting that with optimized implantation technique, LBBB may also be mitigated, although the substantial heterogeneity in this subgroup warrants cautious interpretation.

The potential synergy between cusp-overlap fluoroscopy and intracardiac echocardiography (ICE) represents an important direction for further procedural refinement. COT optimizes the fluoroscopic projection by isolating the non-coronary cusp and improving visualization of the left ventricular outflow tract, thereby facilitating a higher and more controlled valve deployment. ICE, in contrast, provides real-time intraprocedural visualization of anatomical landmarks relevant to conduction injury, particularly the membranous septum. Because the interaction between membranous septum length and implantation depth is a key determinant of post-TAVR conduction disturbance and permanent pacemaker implantation, combining COT with ICE may enable a more individualized implantation strategy tailored to each patient's septal anatomy. In this multimodality framework, fluoroscopy guides optimal device orientation, whereas ICE helps define a patient-specific safe implantation depth relative to the conduction system. Ishizu et al. reported that transjugular ICE-guided self-expandable TAVR using COT was associated with higher valve positioning and lower rates of conduction disturbances and PPI, supporting the biological plausibility of this strategy. However, this concept remains investigational, and prospective multicenter studies are needed before ICE-guided tailored implantation can be recommended as a standard approach (12, 42, 52, 53).

Importantly, the reduction in conduction injury was accompanied by a measurable mortality benefit, largely driven by early follow-up outcomes. Although mortality after TAVR is multifactorial, avoidance of pacemaker implantation and high-grade conduction abnormalities may improve short-term recovery through prevention of ventricular dyssynchrony, preservation of ventricular function, and shorter hospitalization. The absence of a consistent long-term mortality difference likely reflects limited follow-up duration and residual confounding inherent to observational studies rather than a lack of a true biological effect. Collectively, these findings indicate that optimization of implantation depth using COT may influence not only procedural endpoints but also clinically meaningful patient outcomes. In contrast, the risk of cerebrovascular accidents was similar between the cusp-overlap and standard three-cusp techniques. Notably, the absence of heterogeneity for this outcome (*I*^2^ = 0%) suggests that the neutral effect on stroke risk was highly consistent across diverse study populations, valve platforms, and clinical settings, supporting the generalizability of this finding. The inclusion of Lee et al. (51) strengthened the PPI and conduction disturbance analyses with additional events; as expected from a study that did not report mortality outcomes, this addition did not influence the pooled mortality estimate, which remained robust and consistent.

In addition to reducing conduction injury, COT may have implications for commissural alignment and long-term valve management. Commissural alignment has become increasingly important as TAVR expands to younger and lower-risk populations with longer life expectancy. Proper alignment of the transcatheter heart valve commissures may reduce overlap between the prosthetic commissural posts and coronary ostia, thereby preserving future coronary access for angiography or percutaneous coronary intervention. This is particularly relevant for self-expandable valves, in which tall frames and supra-annular leaflet position may complicate coronary re-engagement if commissural orientation is unfavorable. Furthermore, favorable commissural alignment is an important procedural consideration for future TAV-in-TAV procedures, because coronary access and avoidance of coronary obstruction are central to lifetime management planning. Thus, COT should not be viewed only as a technique for reducing PPI, but also as part of a broader procedural strategy aimed at preserving future therapeutic options across the patient's lifetime (11, 12, 54).

By mitigating one of the principal limitations of self-expandable valves, namely the higher risk of conduction disturbance and PPI, COT may increase the attractiveness of SEVs in broader patient populations, including younger and lower-risk patients in whom supra-annular hemodynamics, larger effective orifice area, and long-term valve performance are important considerations. However, our analysis was not designed to compare valve platforms directly, and this interpretation should therefore be considered hypothesis-generating.

Importantly, publication-bias adjustment using the trim-and-fill method did not materially alter the observed reductions in all-cause mortality and permanent pacemaker implantation. Both outcomes remained statistically significant after adjustment, supporting the robustness of these associations despite the observational nature of the available evidence. In contrast, the adjusted estimate for conduction disturbances was attenuated and became statistically non-significant, suggesting reduced certainty and possible small-study effects rather than definitive absence of benefit. These findings collectively indicate that the strongest evidence supporting the cusp-overlap technique relates to its impact on mortality and permanent pacemaker implantation.

Our risk of bias assessment using the ROBINS-I tool revealed that no study achieved an overall low risk of bias, with six studies rated as moderate, ten as serious, and two as critical (35, 51). The predominant limitation was bias due to confounding, reflecting the non-randomized, before-and-after designs employed by all included studies. Lee et al. (51) was rated as critical risk primarily due to confounding by valve generation (Evolut R vs. Evolut PRO) and the use of 1-week rather than 30-day endpoints, while Sztejfman et al. (35) was rated as critical due to extreme group imbalance and imprecise effect estimates. Nonetheless, sensitivity analyses restricted to the six studies with moderate risk of bias yielded directionally consistent results, supporting the reliability of our primary findings.

Recent comparative evidence evaluating the cusp-overlap technique is generally consistent with the direction of effect observed in our analysis. The 2025 meta-analysis by Khalefa et al. (55) reported a borderline reduction in 30-day mortality and a significant decrease in permanent pacemaker implantation with COT compared with the standard three-cusp view. Our findings extend these observations by demonstrating a statistically significant reduction in overall mortality and a more pronounced reduction in PPI, as well as a marked reduction in high-grade atrioventricular block and a newly observed reduction in LBBB. The stronger and more comprehensive signal in our pooled analysis may reflect the larger aggregated population (over 7,200 patients) and the inclusion of more contemporary cohorts, including Lee et al. (51), using optimized implantation strategies. Importantly, both analyses consistently showed no increase in procedural complications, reinforcing that higher implantation depth achieved with COT improves electrical outcomes without compromising safety.

The clinical relevance of these electrical benefits is further supported when interpreted in the context of prior literature on post-TAVR conduction injury. Earlier meta-analyses and cohort studies, including Faroux et al. and Fadahunsi et al. (52, 53), demonstrated that permanent pacemaker implantation and persistent conduction abnormalities after TAVR are associated with increased mortality and heart-failure hospitalization during follow-up. Similarly, broader systematic reviews such as Van Rosendael et al. (54) emphasized that procedural factors strongly influence pacemaker risk across valve platforms. Therefore, the reduction in pacemaker implantation and complete atrioventricular block observed in our study provides a plausible mechanistic explanation for the mortality benefit seen with COT. Rather than representing a direct survival effect of the fluoroscopic projection itself, our results suggest that optimizing implantation depth mitigates conduction system injury, which in turn may translate into improved early clinical outcomes after self-expandable TAVR.

### 4.1 Limitations of the study

Several limitations should be considered when interpreting the findings of this meta-analysis. First, all included studies were observational and non-randomized, making the results inherently susceptible to residual confounding despite adjustment strategies employed in individual investigations. Important clinical and procedural variables, including baseline conduction abnormalities, implantation depth, valve sizing strategy, annular and left ventricular outflow tract anatomy, degree of calcification, and center-specific procedural protocols, may not have been uniformly measured or adequately controlled across studies. Consequently, the observed associations between the cusp-overlap technique and clinical outcomes cannot be interpreted as definitive evidence of causality.

Second, selection bias cannot be excluded because treatment allocation was not randomized. In most studies, the choice of fluoroscopic projection was determined by operator preference, institutional practice patterns, or temporal adoption of the cusp-overlap technique. Therefore, patients treated with cusp-overlap-guided implantation may have differed systematically from those treated with the standard three-cusp approach in ways that could influence procedural and clinical outcomes.

Third, operator experience and learning-curve effects may have influenced the reported outcomes. The cusp-overlap technique has been progressively adopted during the evolution of contemporary TAVR practice, and improved procedural results may partially reflect increasing operator expertise, advances in preprocedural planning, and refinement of deployment techniques rather than the fluoroscopic projection strategy alone. Similarly, temporal improvements in TAVR technology, including newer-generation self-expanding valve systems, enhanced imaging modalities, and procedural standardization, may have contributed to improved outcomes in more recent studies independent of the cusp-overlap technique itself.

Fourth, differences in baseline patient characteristics among studies may have introduced additional sources of variability. Variations in age, comorbidity burden, surgical risk profile, anatomical complexity, prevalence of pre-existing conduction disturbances, and indications for TAVR may have influenced outcome estimates. Because the present analysis was based on aggregate study-level data, detailed adjustment for these patient-level factors was not possible.

Fifth, moderate-to-substantial between-study heterogeneity was observed for several outcomes, particularly conduction disturbances, reflecting differences in study design, patient populations, valve platforms, procedural techniques, endpoint definitions, and follow-up duration. Although random-effects models were used to account for statistical heterogeneity, residual clinical and methodological heterogeneity may still affect the precision and generalizability of the pooled estimates.

Sixth, although formal publication-bias assessments did not consistently demonstrate significant asymmetry, the possibility of small-study effects and selective reporting cannot be completely excluded. This concern is particularly relevant for outcomes evaluated in a limited number of studies and may have influenced the magnitude of the observed treatment effects.

Furthermore, fluoroscopic projection optimization alone cannot fully eliminate the risk of post-TAVR conduction disturbances. Patient-specific and device-related factors, including baseline conduction abnormalities, membranous septum length, implantation depth, annular and LVOT calcification burden, valve oversizing, and device platform characteristics, remain important determinants of conduction-system injury and permanent pacemaker implantation. Therefore, COT should be considered one component of a comprehensive implantation strategy rather than a universal solution for preventing conduction complications.

Finally, according to the GRADE framework, the certainty of evidence ranged from very low to low across the evaluated outcomes because of the observational nature of the included studies, risk of bias, heterogeneity, and potential imprecision. Therefore, while the observed reductions in conduction disturbances, permanent pacemaker implantation, and mortality are clinically encouraging and biologically plausible, these findings should be interpreted cautiously. Large, adequately powered randomized controlled trials and individual-patient-data analyses are needed to confirm these results, better characterize treatment-effect modifiers, and establish the role of cusp-overlap-guided implantation in routine contemporary TAVR practice.

### 4.2 Recommendations for future research

Future research should focus on generating higher-quality comparative evidence. Randomized controlled trials or prospective multicenter registries with standardized endpoint definitions are needed to establish causality and better quantify the clinical benefit of the cusp-overlap technique. In addition, individual participant data meta-analyses would allow more accurate adjustment for confounders and enable time-to-event analyses to clarify long-term outcomes. Standardization of conduction disturbance definitions, pacemaker indications, and reporting time points will also be essential to reduce heterogeneity across studies. Mechanistic investigations incorporating advanced imaging and procedural measurements are warranted to directly link implantation depth, anatomical characteristics, and conduction injury. Future studies should also evaluate effect modification according to valve platform, device generation, annular and left ventricular outflow tract calcification, and pre-existing conduction abnormalities to identify patient subgroups most likely to benefit. Finally, longer-term follow-up studies are required to determine whether reductions in pacemaker implantation translate into improved heart-failure outcomes, quality of life, and cost-effectiveness. Research addressing operator learning curves and standardized training strategies will further support safe and reproducible implementation of the cusp-overlap technique in routine clinical practice.

## 5 Conclusion

In patients undergoing self-expandable transcatheter aortic valve replacement (TAVR), the cusp-overlap technique was associated with significantly lower rates of conduction disturbances and permanent pacemaker implantation compared with the standard three-cusp coplanar approach. In addition, a reduction in all-cause mortality was observed, while the risk of cerebrovascular events appeared similar between strategies. These findings are biologically plausible and support the concept that optimization of implantation depth through cusp-overlap-guided deployment may improve procedural and clinical outcomes. However, the available evidence is derived exclusively from observational studies and remains subject to residual confounding, selection bias, heterogeneity in study design and outcome definitions, and potential publication bias. Consequently, the overall certainty of evidence remains low, and the observed associations should not be interpreted as definitive evidence of causality. Taken together, the current evidence suggests that the cusp-overlap technique represents a promising procedural refinement in contemporary self-expandable TAVR practice, particularly for reducing conduction-system injury and permanent pacemaker implantation. Nevertheless, adequately powered randomized controlled trials and prospective multicenter studies are required to confirm these findings, establish long-term clinical benefits, and define the optimal role of cusp-overlap-guided implantation in routine clinical practice.

## Data Availability

The original contributions presented in the study are included in the article/Supplementary Material, further inquiries can be directed to the corresponding author.
