# Supplementary material for: Systematic review, meta-analysis of cusp-overlap vs. three-cusp coplanar approaches in self-expandable transcatheter aortic valve replacement
Source: Front Cardiovasc Med. 2026 Jul 2;13:1870774. doi: 10.3389/fcvm.2026.1870774 (PMC13373035; doi:10.3389/fcvm.2026.1870774)
Supplement: Supplementary file 1 [file Supplementaryfile1.docx]

## Search and Screening

*Appendix Table 1. Detailed Search Strategy Across Databases*

| **Database** | **Search Strategy** |
| --- | --- |
| **PubMed (MEDLINE)** | (((("Transcatheter Aortic Valve Replacement"[Mesh]) OR TAVR OR TAVI OR "transcatheter aortic valve replacement" OR "transcatheter aortic valve implantation")) AND ((("Aortic Valve Stenosis"[Mesh]) OR "aortic stenosis" OR "severe aortic stenosis" OR "aortic valve disease")) AND (("cusp overlap") OR "cusp-overlap" OR "cusp overlap technique" OR "cusp-overlap view" OR "overlap view" OR "cusp-overlapping projection")) AND (("three cusp") OR "three-cusp" OR "three cusp view" OR "coplanar view" OR "standard view")) AND (("self-expandable") OR "self-expanding" OR "self expandable valve" OR "self expanding valve" OR Evolut OR CoreValve OR Portico)) |
| **Embase** | ('transcatheter aortic valve implantation'/exp OR 'transcatheter aortic valve replacement' OR tavr OR tavi) AND ('aortic stenosis'/exp OR 'aortic stenosis' OR 'aortic valve disease') AND ('cusp overlap' OR 'cusp overlap technique' OR 'cusp overlap view' OR 'overlap view') AND ('three cusp view' OR 'three-cusp view' OR 'coplanar view') AND ('self expandable valve' OR 'self expanding valve' OR evolut OR corevalve OR portico) |
| **Scopus** | TITLE-ABS-KEY(("transcatheter aortic valve replacement" OR "transcatheter aortic valve implantation" OR TAVR OR TAVI) AND ("aortic stenosis" OR "aortic valve disease") AND ("cusp overlap" OR "cusp-overlap" OR "cusp overlap technique" OR "overlap view") AND ("three cusp" OR "three-cusp" OR "coplanar view") AND ("self-expandable" OR "self-expanding" OR Evolut OR CoreValve OR Portico)) |
| **Web of Science Core Collection** | TS=(("transcatheter aortic valve replacement" OR "transcatheter aortic valve implantation" OR TAVR OR TAVI) AND ("aortic stenosis" OR "aortic valve disease") AND ("cusp overlap" OR "cusp-overlap" OR "overlap view") AND ("three cusp" OR "three-cusp" OR "coplanar view") AND ("self-expandable" OR "self-expanding" OR Evolut OR CoreValve OR Portico)) |
| **Cochrane Library** | ("transcatheter aortic valve replacement" OR TAVR OR TAVI OR "transcatheter aortic valve implantation") AND ("aortic stenosis" OR "aortic valve disease") AND ("cusp overlap" OR "cusp-overlap" OR "overlap view") AND ("three cusp" OR "three-cusp" OR "coplanar view") AND ("self-expandable" OR "self-expanding" OR Evolut OR CoreValve OR Portico) |
